# The Potassium Channel Blocker β-Bungarotoxin from the Krait *Bungarus multicinctus* Venom Manifests Antiprotozoal Activity

**DOI:** 10.3390/biomedicines11041115

**Published:** 2023-04-07

**Authors:** Alexey V. Osipov, Elena G. Cheremnykh, Rustam H. Ziganshin, Vladislav G. Starkov, Trang Thuy Thi Nguyen, Khoa Cuu Nguyen, Dung Tien Le, Anh Ngoc Hoang, Victor I. Tsetlin, Yuri N. Utkin

**Affiliations:** 1 Shemyakin-Ovchinnikov Institute of Bioorganic Chemistry, Russian Academy of Sciences, Moscow 117997, Russia; 2 Mental Health Research Centre, Moscow 115522, Russia; 3 Faculty of Pharmacy, Nguyen Tat Thanh University, Ho Chi Minh City 700000, Vietnam; 4 Institute of Applied Materials Science, Vietnam Academy of Science and Technology, Ho Chi Minh City 700000, Vietnam

**Keywords:** antiprotozoal activity, β-bungarotoxin, ciliate, krait, potassium channel, venom

## Abstract

Protozoal infections are a world-wide problem. The toxicity and somewhat low effectiveness of the existing drugs require the search for new ways of protozoa suppression. Snake venom contains structurally diverse components manifesting antiprotozoal activity; for example, those in cobra venom are cytotoxins. In this work, we aimed to characterize a novel antiprotozoal component(s) in the *Bungarus multicinctus* krait venom using the ciliate *Tetrahymena pyriformis* as a model organism. To determine the toxicity of the substances under study, surviving ciliates were registered automatically by an original BioLaT-3.2 instrument. The krait venom was separated by three-step liquid chromatography and the toxicity of the obtained fractions against *T. pyriformis* was analyzed. As a result, 21 kDa protein toxic to *Tetrahymena* was isolated and its amino acid sequence was determined by MALDI TOF MS and high-resolution mass spectrometry. It was found that antiprotozoal activity was manifested by β-bungarotoxin (β-Bgt) differing from the known toxins by two amino acid residues. Inactivation of β-Bgt phospholipolytic activity with *p*-bromophenacyl bromide did not change its antiprotozoal activity. Thus, this is the first demonstration of the antiprotozoal activity of β-Bgt, which is shown to be independent of its phospholipolytic activity.

## 1. Introduction

Protozoa are a diverse group of single-cell eukaryotic organisms. Parasitic protozoa are causative agents of life-threatening diseases in humans (e.g., malaria, amoebiasis, Chagas disease, etc.) and of dangerous diseases specific for domestic and wild animals. As protozoa are eukaryotes and share a number of biochemical and metabolic pathways with chordates, most antibacterial drugs are ineffective in the treatment of protozoal infections. Currently, there is a number of antiprotozoal drugs, but all of them have significant side effects (see for instance, [1]). In addition, effective treatment is hindered by the development of drug resistance in pathogens [2]. The toxicity of the drugs, duration of treatment, and sometimes their low effectiveness stimulate the search for new ways of protozoa suppression. Antiprotozoal compounds with the desired properties may be found in animal venoms [3].

Snake venom is a pool of bioactive peptides and proteins targeting vital systems of a prey. Due to their inherent high affinity and selectivity, many venom components are used as versatile biochemical tools or considered as prototypes of medicines. The majority of snake preys are vertebrates and the most obvious and so the most studied effects of snake venoms and of their components are on vertebrates. There are also some data on the toxicity of snake venoms to invertebrates, mainly worms [4] and insects [5], which serve as prey for some snake species. In addition, there are effects of snake venoms on microorganisms. Thus, the venoms can exhibit antiviral (reviewed in [6]), antibacterial (reviewed in [7]), and antiprotozoal activities (reviewed in [3,8]). By the present time, snake venom peptides and proteins with such activities have been identified. For instance, cathelicidins possess antibacterial and anti-fungal properties [9], while phospholipases A2 may exert antibacterial [10] and antiviral ones [11].

So far, antiprotozoal activity has been found in snake venoms from various families and genera, including Asian elapids, as well as Asian, African and American viperids, and Colubrid [3,8]. Together with recent findings [12,13,14,15], these data suggest that antiprotozoal activity may be a widespread property of snake venom.

The types of toxins in snake venoms possessing antiprotozoal activity are quite diverse; even venoms of snake species from one genus may contain different antiprotozoal compounds. Moreover, the venom of a single species may contain antiprotozoal toxins from different protein families. Thus, the antiprotozoal toxins identified so far are enzymes including L-amino acid oxidases from the venoms of *Bothrops* spp. [16], *Lachesis muta* [17], and *Calloselasma rhodostoma* [18], phospholipases A2 from the venoms of *Crotalus* and *Bothrops* spp. [8], *Naja nigricollis* [19] and some other species, as well as metalloproteases from the venoms of *B. neuwiedi* [20] and *B. moojeni* [21]. Non-enzymatic toxins include a lectin from *B. leucurus* venom [22], a disintegrin from *Cerastes cerastes* venom [23], a small pore-forming myotoxin crotamine from *Crotalus* venom [24], cathelicidin-like proteins crotalicidin and batroxicidin from *C. durissus terrificus* and *B. atrox* venoms [25,26], a CRiSP crovirin from *C. viridis* [27], and cytotoxins from cobra venoms [14]. It should be noted that the antiprotozoal activity of snake toxins may partially depend on the species of protozoa studied, although such data have not yet been systematized. Thus, the nature of the antiprotozoal components of snake venom is quite diverse, and some of them might serve as the basis for the development of appropriate drugs.

The protozoa group is very diverse and includes both pathogenic and non-pathogenic organisms. In this work, we used infusorian *T. pyriformis* as a model for the study of antiprotozoal activity. The infusoria are taxonomically fairly far from several important protozoan parasites, including *Trypanosoma* spp., *Leishmania* spp., and *Plasmodium* spp. However, in our opinion, the protozoa are characterized by a number of common features, which have allowed for us to use *Tetrahymena* as a model organism. A common feature of most protozoan parasites is the locomotion in the surrounding medium. The protozoans are traditionally divided, based on their mode of locomotion, into flagellates, amoeboids, sporozoans and ciliates. Motility is especially important for their ability to detect and enter target cells or organs of their hosts; therefore, it may represent a new object for chemotherapy. The important role in parasite pathology, transmission, survival, and proliferation is played by the motility of the single or multiple flagella [28]. Flagellar motility is observed in various parasitic protozoans. For example, a bloodstream form of trypanosomes swims rapidly to remove surface-bound antibodies and escape host attack [29]. Flagellar motility is necessary for the transmission of *Leishmania* parasites [30]. Ciliary motility is absolutely necessary for the survival of ciliates, which include *Tetrahymena corlissi*, a parasite in freshwater fishes [31] and *Balantidium coli* known to be pathogenic to humans. A feature of *T. pyriformis*, like many other ciliates, is their continuous movement, which is regulated by ion channels; when these channels are blocked, the cells stop and die [32]. In parasite protozoa, ion channels also play an important physiological role as regulators of cellular functions and host–parasite interactions [33]. However, the molecular mechanisms of ciliary and flagellar motility are quite similar [34]; therefore, the compounds affecting ciliary motility of *Tetrahymena* can influence the flagellar motility of other protozoa, including pathogenic *Trypanosoma* and *Leishmania*. So, in this respect, *Tetrahymena* can be considered as an appropriate model of pathogenic protozoa.

In some protozoa, a cytoskeletal infrastructure, which is called a “pellicle”, is located under the outer membrane of the cell. The pellicle preserves the shape of the cells, especially during movement, and plays many other important roles by providing a barrier between the inside and outside of the cell, taking part in the regulation of ion fluxes and in the transportation of vesicles with food and waste in and out, etc. Pellicle is characteristic for both non-pathogenic and pathogenic protozoan. So far, the pellicular composition of ciliates has received more scientific attention, and among the human parasites, the pellicles of *Plasmodium* and *Toxoplasma* have been most examined [35]. Some data about the pellicle were reported for pathogenic *Leishmania* [36] and *Trypanosoma* [37]. Since pellicles are located at the cell surface, it is easily exposed to external agents including antiprotozoal drugs. The damage of the pellicle results in cell death [38]. As a typical protozoan with pellicle, *Tetrahymena* is an adequate model for the discovery of agents acting on this organelle in pathogenic cells.

Some ciliates, including *Paramecium caudatum*, *T. pyriformis*, and *Oxytricha trifallax*, are model organisms for molecular biology. The ciliary protozoa *Tetrahymena* spp., extremely motile free-living organisms [39], has long been used in environmental studies to determine the cytotoxicity of environmental pollutants and chemicals, as well as in toxicology as a model organism [40]. Because *Tetrahymena* as a model species is not demanding on habitat conditions and reproduce easily, it is simple to grow in large quantities in the laboratory and is well suited for automating cytotoxicity experiments [41].

We previously used ciliates *T. pyriformis* as a model organism for screening snake venoms to search for compounds with antiprotozoal activity [14,15] and found that the cobra venoms were the most toxic among them. They were followed by krait venoms. We found that cytotoxins were responsible for the strong antiprotozoal activity of *N. oxiana* and *N. sumatrana* cobra venoms [14]. However, krait venom does not contain cytotoxins. Moreover, phospholipases A2 and L-amino acid oxidase are also unlikely to be the key antiprotozoal components in krait venoms because the *B. multicinctus* venom is about 20 times more active than the venom of krait *B. fasciatus* [15], while the content of phospholipases A2 and L-amino acid oxidase is significantly higher in *B. fasciatus* venom [42]. Therefore, there should be a different type of compound responsible for the antiprotozoal activity of the *B. multicinctus* venom. The question arises: what is this compound? To answer it, the following goals were set in this work:

- To isolate this compound in pure form;
- To determine its antiprotozoal activity;
- To establish its amino acid sequence;
- To reveal functional characteristics essential for its antiprotozoal activity.

We found that it is β-bungarotoxin, thus providing the first indication of the antiprotozoal activity for this class of toxins.

## 2. Materials and Methods

### 2.1. Materials

The *B. multicinctus* krait venom was obtained from the authorized, licensed farm for snake breeding and venom production located in Vinh Shon (Vinh Tuong, Vinh Phuc province, Vietnam). The certificate of farm has been registered in accordance with the Government’s Decree No. 82/2006/ND-CP of 10 August 2006 by the Department of Agriculture and Rural Development of the Socialist Republic of Vietnam. The venom obtained was dried over anhydrous CaCl_2_ and stored at −20 °C until use.

The 4-Bromophenacyl bromide was from Lancaster Synthesis (Morecambe, UK). The 1-Hexadecanoyl-2-(1-pyrenedecanoyl)-*sn*-glycero-3-phosphocholine was from Invitrogen Molecular Probes (Eugene, OR, USA). Pancreatic casein hydrolysate and trifluoroacetic acids were from Merck KGaA (Darmstadt, Germany), Springer 0251 yeast extract from Bio Springer (Maisons-Alfort, France). Acetonitrile was purchased from Catrosa Reaktiv LLC (Moscow, Russia). All other reagents of analytical grade or higher purity were from local Russian suppliers.

### 2.2. Cultivation of Tetrahymena pyriformis 

Infusoria *T. pyriformis* (strain WH14) was obtained from the collection of the All-Russian Research Institute of Veterinary Sanitation, Hygiene and Ecology (Moscow, Russia). Cultivation of infusoria was carried out under aseptic conditions in a medium containing tryptone (10 g/L), yeast extract (2 g/L), glucose (5 g/L) and NaCl (1 g/L). This solution was poured into test tubes with cotton-gauze stoppers, 4 mL in each, and sterilized in an autoclave at 0.5 atm for 30 min. After medium cooling, infusoria were seeded with a transfer loop above the burner flame from the mother liquor into the tubes. The tubes with cell culture were kept in a thermostat at a constant temperature of 25 °C. After 4–5 days of seeding, the infusoria were used for the studies as test organisms. The cells were observed using stereoscopic binocular microscope MBS-10 (JSC LZOS, Moscow region, Russia).

### 2.3. Measurement of Toxicity for Tetrahymena pyriformis 

Toxicity of the venom and its fractions to *T. pyriformis* were measured using a BioLaT-3.2 instrument operating under the AutoCiliataXP program (Europolytest Ltd., Moscow, Russia) essentially, as described [15]. In brief, infusoria was cultivated in the medium containing 0.5% pancreatic casein hydrolysate (Merck KGaA, Darmstadt, Germany), 0.5% glucose, 0.1% Springer 0251 yeast extract (Bio Springer, Maisons-Alfort, France) and 0.1% NaCl. For the toxicity measurements, the tested solutions (5–50 µL) were added to the suspension of *T. pyriformis* cells in measuring wells of the instrument, and the number of survived cells was counted each minute using the AutoCiliataXP program. Each solution was measured in 3 wells. Only the number of moving cells at each minute was counted. The real-time dependences of the number of survived cells on the time were measured. In a short-term experiment (up to 1 h), 2000 to 4000 cells were used. In a long-term experiment, 290 µL of the aqueous solutions of toxins was added to the wells of the instrument, the AutoCiliata program was switched on, and the samples were placed in the instrument. After the wells with samples without ciliates were evaluated by the program, a suspension of ciliates containing 500–1000 cells was added to each well. The final sample volume was 300 µL. Each measurement was carried out in triplicate. Then, the cells were counted ten-fold and the results were recorded. The samples were placed in a thermostat, and after 24 h, the ciliates were counted again using the program.

For quantification, survival rates (K) were calculated:

K = A_24_/A_0_,

where A_24_ is the number of live ciliates in the sample after 24 h of exposure and A_0_ is the number of live ciliates at the beginning of the experiment. In the control experiments, with a 24 h exposure of ciliates in distilled water, this rate was in the range from 2 to 2.5. To determine the sensitivity of protozoa, a solution of CuSO_4_ (0.1 μM) was used as a positive control—it produced the death of all cells within one hour. 

### 2.4. Venom Fractionation and Isolation of Active Compound

**Figure 1:**
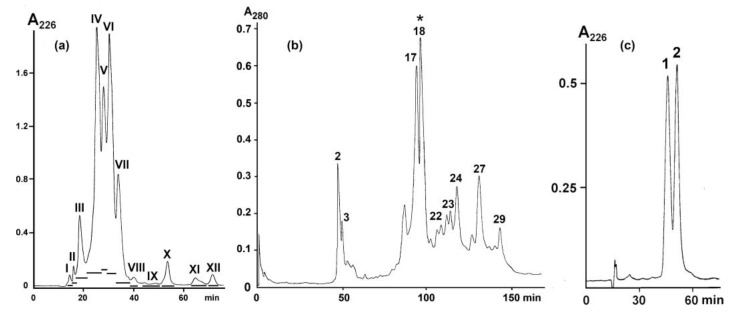
Separation of *B. multicinctus* venom. (**a**) Separation of the crude venom by gel-filtration chromatography on Superdex 75 column. (**b**) Separation of active fraction IV using ion-exchange chromatography on HEMA 1000 CM column. The asterisk indicates the active fraction. (**c**) Fraction 18 was separated into individual components by RP-HPLC using Jupiter C18 column.

Crude *B. multicinctus* venom (100 mg) was fractionated by gel filtration using Superdex 75 column (10 × 300 mm, GE Healthcare, Chicago, IL, USA) in 0.1 M ammonium acetate buffer, pH 6.2 at a flow rate of 0.5 mL/min. The fractions obtained (Figure 1a) were screened for the toxicity to *T. pyriformis*. The active fraction IV was separated by ion-exchange chromatography on HEMA 1000 CM column (8 × 250 nm, Tessek Ltd., Prague, Czech Republic) using a gradient of ammonium acetate concentration from 5 mM to 0.8 M (pH 7.5) in two steps—from 5 mM to 0.4 M in 120 min and from 0.4 to 0.8 M in 49 min. The toxicity measurement of the fractions obtained (Figure 1b) revealed that the most active was fraction 18. It was further separated by RP-HPLC on Jupiter C18 column (10 × 250 mm, Phenomenex, Torrance, CA, USA) using a gradient of acetonitrile concentration (from 27 to 35% in 60 min) in the presence of 0.1% trifluoroacetic acid (Figure 1c).

### 2.5. Mass Spectrometry 

#### 2.5.1. MALDI-TOF Mass Spectrometry 

MALDI-TOF mass spectrometry (MS) was performed as described [43] using Ultraflex-TOF/TOF mass spectrometer (Bruker Daltonics, Bremen, Germany).

#### 2.5.2. High Resolution Mass Spectrometry

LC-MS analysis of protein from the fraction IV-18-2 was performed with an Ultimate 3000 Nano LC System (Thermo Fisher Scientific, Waltham, MA, USA), which was coupled to the Q Exactive Plus mass spectrometer (Thermo Fisher Scientific, Waltham, MA, USA) via a nanoelectrospray source (Thermo Fisher Scientific, Waltham, MA, USA). Protein fraction was separated in a home-packed fused-silica column 300 × 0.1 mm packed with Reprosil PUR C18AQ 1.9 (Dr. Maisch HPLC GmbH, Ammerbuch, Germany) into an emitter prepared with P2000 Laser Puller (Sutter Instrument, Novato, CA, USA). Protein fraction was loaded in a loading solution (98% 0.1% (*v/v*) formic acid, 2% (*v/v*) acetonitrile) and eluted with a linear gradient: 10–60% B for 15 min at a flow rate of 500 nL/min. MS1 parameters were as follows: 140K resolution, 500–2000 scan range, max injection time—200 ms, AGC target—3 × 10^6^.

Protein amino acid sequencing by LC-MS/MS was performed as follows: Protein from the fraction IV-18-2 was reduced, carbamidomethylated and hydrolyzed either with trypsin or endoproteinase Glu-C. Peptides were separated on a 250 mm 75-µm inner diameter Thermo Scientific^TM^ Acclaim™ PepMap™ 100 C18 LC column with particle size 3 µm. Reverse-phase chromatography was performed with an Ultimate 3000 Nano LC System (Thermo Fisher Scientific, Waltham, MA, USA), which was coupled to the Q Exactive HF mass spectrometer (Thermo Fisher Scientific, Waltham, MA, USA) via a nanoelectrospray source (Thermo Fisher Scientific, Waltham, MA, USA). Approximately 1 µg of trypsin digest was applied to the column in buffer A (0.2% (*v/v*) formic acid) and then eluted with a linear gradient of 4–55% buffer B (0.1% (*v/v*) formic acid, 80% (*v/v*) acetonitrile) in 120 min at a flow rate of 350 nL/min. After each separation, the column was washed for 5 min with 95% buffer B and re-equilibrated for 5 min with buffer A. The temperature of the column was maintained at 40 °C. An automatic switch between a full scan and up to 15 data-dependent MS/MS scans (method topN) was used for MS data acquisition. The target value for the full scan MS spectra was 3 × 10^6^ in the 300−1200 *m/z* range with a maximum injection time of 30 ms and a resolution of 60,000. A 1.4 *m/z* window and a fixed first mass of 100.0 *m/z* were applied for the isolation of precursors. Higher-energy dissociation (HCD) with a normalized collision energy of 28 eV was used for the precursor’s fragmentation. A resolution of 15,000 at *m/z* 400 with an ion target value of 1 × 10^5^ in the 200−2000 *m/z* range with a maximum injection time of 60 ms were used for MS/MS scans acquisition. The selected peptide candidates were excluded for 30 s to minimize the repeat sequencing of peptides.

MS raw files were analyzed by Peaks studio 10.0 (Bioinformatics Solutions Inc., Waterloo, ON, Canada) [44]. The identification of proteins was made by searching against the NCBI database of protein sequences from Serpentes (version from 06.2016, containing 162,874 entry) with a deamidation Asn/Gln and Met oxidation as variable modifications and carbamidomethyl Cys as a fixed modification. The false discovery rate for peptide-spectrum matches was set to 0.01 as determined by searching a reverse database. A maximum of two missed cleavages were allowed in the database search. Trypsin specificity was set as C-terminal to lysine and arginine and Glu-C specificity as C-terminal to aspartic and glutamic acids. An allowed initial precursor mass deviation up to 10 p.p.m. and an allowed fragment mass deviation 0.02 Da were applied for peptide identification. 

### 2.6. Modification of β-Bgt with p-Bromophenacyl Bromide

To the solution of 440 µg of the toxin in 2 mL of 0.025 mM Tris-HCl buffer (pH 8.1), 100 µL of the solution of 4-bromophenacyl bromide (4 mg/mL) in acetone was added and the reaction mixture was incubated with strong shaking for 3 h at a room temperature. The reaction products were separated on a Jupiter C18 column (10 × 250 mm, Phenomenex, Torrance, CA, USA) in a gradient of acetonitrile in water from 15 to 55% in 40 min in the presence of 0.1% TFA at a flow rate of 1.8 mL/min. The fractions obtained were freeze-dried and used for further study. Phospholipolytic activity was determined by a fluorometric assay according to [45]. Briefly, 2 µL of the solution of toxin or its derivative (2 mg/mL) was added to 1 mL of 50 mM Tris-HCl buffer, pH 7.5, containing 100 mM NaCl, 1 mM EDTA, 6 mM CaCl_2_, 0.1% bovine serum albumin, and 2 μM 1-hexadecanoyl-2-(1-pyrenedecanoyl)-sn-glycero-3-phosphocholine as a substrate. Fluorescence intensity was monitored using Hitachi F-400 spectrofluorometer (Hitachi, Tokyo, Japan) at excitation and emission wavelengths of 345 and 398 nm, respectively.

### 2.7. Influence of Tetraethylammonium on T. pyriformis Cells

The measurements were carried out as described in Section 2.3. Thetraethylammonium chloride at a concentration of 100 mM was added instead of the venom fractions. 

### 2.8. Statistical Analysis

Statistical analysis of the data was performed using the Microsoft Excel 2019 statistical software package. Data are presented as means ± relative standard deviation (coefficient of variation, CV), which is equal to the ratio of the standard deviation to the mean. For each experiment, 3 independent measurements were performed.

## 3. Results

### 3.1. Isolation of Active Compound

As discussed above, our previous studies demonstrated that venoms of different snake species killed a protozoa *T. pyriformis* with varying effectiveness [15]. Cobra venom was the most toxic followed by the venom of krait *B. multicinctus*. As cobra venom was studied by us earlier [14], we chose *B. multicinctus* venom to search for a new antiprotozoal compound(s).

**Figure 2:**
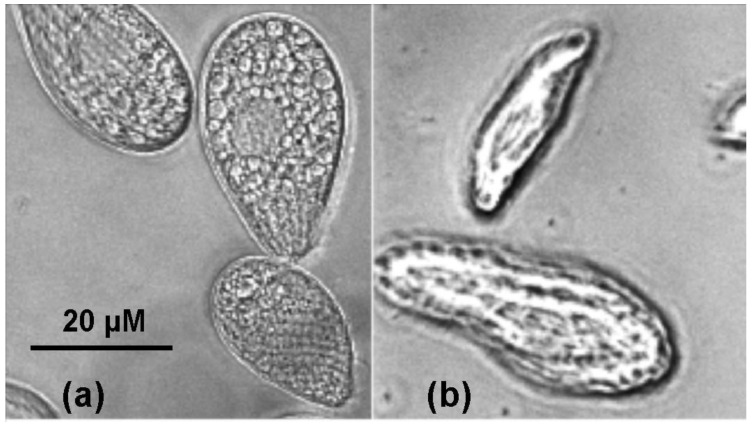
Images of *T. pyriformis* cells. The alive (**a**) and dead (**b**) cells are shown. The images were obtained using MBS-10 microscope at a magnification of 56.

To isolate the active component, a three-stage separation scheme was used. It included gel-filtration as a first stage followed by ion exchange chromatography with final purification by reversed-phase chromatography (Figure 1). At each stage, all the fractions obtained were screened for their toxicity to *Tetrahymena* (Figure 2). 

**Figure 3:**
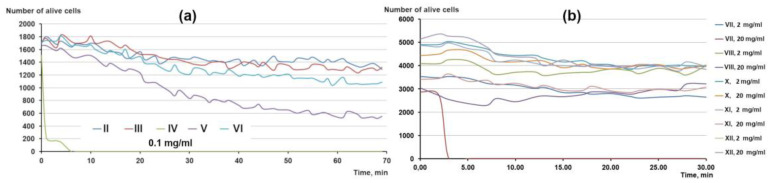
Toxicity to *Tetrahymena* of the fractions obtained by gel-filtration to *Tetrahymena*. (**a**) Activity of fractions I-VI at concentration of 0.1 mg/mL. (**b**) Activity of fractions VII-XII at concentrations of 2 and 20 mg/mL.

After gel-filtration, 12 fractions were obtained (Figure 1a). Antiprotozoal activity determination showed fraction IV as the most active (Figure 3). At a concentration of 0.1 mg/mL, it killed all infusoria within less than 10 min (Figure 3a). Lower activity was demonstrated by fraction V, which decreased the number of alive cells by three times within one hour. Some toxicity was observed for fraction VII, which at a concentration of 20 mg/mL, killed all cells within 5 min. However, this fraction exerted no effect at a concentration of 2 mg/mL (Figure 3b). Based on these data, fraction IV was chosen for further separation.

**Figure 4:**
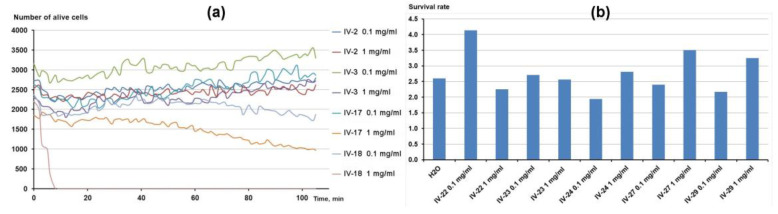
Toxicity to *Tetrahymena* of the fractions obtained by ion-exchange chromatography. (**a**) Activity determination in short-time experiment. (**b**) Activity of fractions at 24 h experiment. Survival rate is the ratio of the number of live ciliates after 24 h of incubation to the number of ciliates at the beginning of the experiment. Three independent measurements were performed for each fraction. For all fractions, the coefficient of variation (CV) did not exceed 0.07, or 7% of the mean.

As krait venoms comprise mostly basic toxins, fraction IV was separated by ion exchange chromatography on a cation exchange column (Figure 1b). As at the previous step, all the fractions obtained were subjected to determination of toxicity to *Tetrahymena* (Figure 4). The initial concentration was 0.1 mg/mL, at which fraction IV was active. If there was no activity at 0.1 mg/mL, a higher concentration of 1 mg/mL was applied. The highest activity was observed for fraction IV-18 (Figure 4a). At a concentration of 1 mg/mL, this fraction killed all the cells within less than 10 min. At 0.1 mg/mL, it decreased the number of alive cells by 20% after 2 h. Lower activity was manifested by fraction IV-17, which at 1 mg/mL, reduced the number of alive cells by about two times (Figure 4a). However, at 0.1 mg/mL, fraction 17 was completely inactive. All other fractions obtained after ion exchange chromatography manifested no toxicity to *Tetrahymena* (Figure 4a,b). Thus, fraction IV-18 was studied further.

Fraction IV-18 was separated by reversed-phase chromatography and two peaks 1 and 2 (Figure 1c) were obtained and named IV-18-1 and IV-18-2, respectively. Interestingly, the separation of fraction IV-17 under the same conditions resulted in a similar profile; the only difference was that peak 1 was higher than peak 2. As fractions IV-17 and IV-18 were not well separated (Figure 1b), we believe that peak 1 corresponds to IV-17 and peak 2 to IV-18. So, we studied further the fraction corresponding to peak 2 (IV-18-2). 

**Figure 5:**
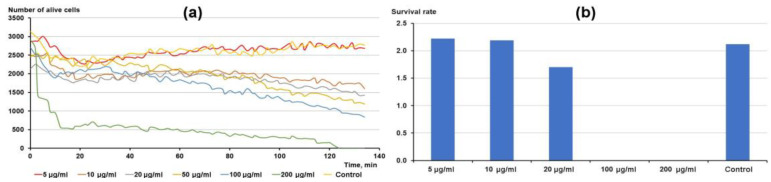
The toxicity of fraction IV-18-2 to *Tetrahymena*. (**a**) Short-time experiment. (**b**) 24 h experiment. Survival rate is the ratio of the number of live ciliates after 24 h of incubation to the number of ciliates at the beginning of the experiment. Three independent measurements were performed for each concentration. For all concentrations, the coefficient of variation (CV) did not exceed 0.07, or 7% of the mean.

Different concentrations of fraction IV-18-2 were used to estimate its specific toxicity (Figure 5). At a concentration of 200 µg/mL, this fraction killed all the cells within 2 h (Figure 4a), while in the 24 h experiment, all cells were dead at 100 µg/mL (Figure 5b). This indicates a high antiprotozoal activity of compound corresponding to fraction IV-18-2. 

### 3.2. Determination of the Structure of Active Protein

To determine the molecular mass and subunit composition of the isolated compound, it was analyzed by polyacrylamide gel electrophoresis under non-reducing and reducing conditions. A single protein band was observed at about 20 kD under non-reducing conditions, while two bands were observed around 14 kDa and 11 kDa after reduction with β-mercaptoethanol. In the *B. multicinctus* venom, β-bungarotoxins (β-Bgt) have a molecular mass of 21 kDa and comprise two polypeptide chains A and B connected by disulfide bond and possessing molecular masses of about 14 kDa and 7 kDa, respectively. So, the analyzed compound may represent β-Bgt with B-chains probably manifesting abnormal electrophoretic mobility. 

The molecular mass and amino acid sequence of the isolated compound (putative β-Bgt) were analyzed by mass-spectrometry (MS). MALDI TOF MS and high-resolution MS (HR MS) were used for the analysis. MALDI-TOF MS revealed the signal corresponding to a molecular mass of about 21 kDa; in addition, the signals corresponding to masses of 7.3 and 13.4 kDa were observed (Table 1). The presence of products with smaller masses can be explained by the MALDI-In Source Decay cleavage of disulfide bonds, which is a well-known phenomenon for MALDI MS [46]. The data of MALDI TOF MS correlate well with the structure of β-Bgt, a molecule of which consists of two subunits with molecular masses similar to those observed. 

**Figure 6:**
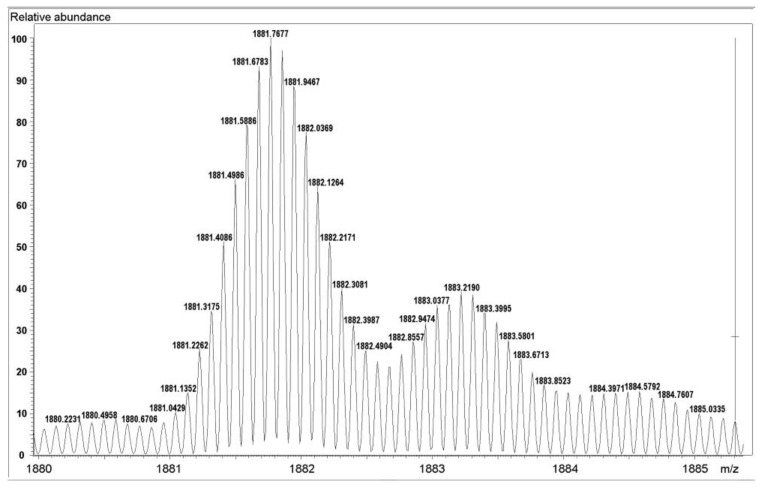
High resolution mass spectrum of compound IV-18-2. z = 11.

**Figure 7:**
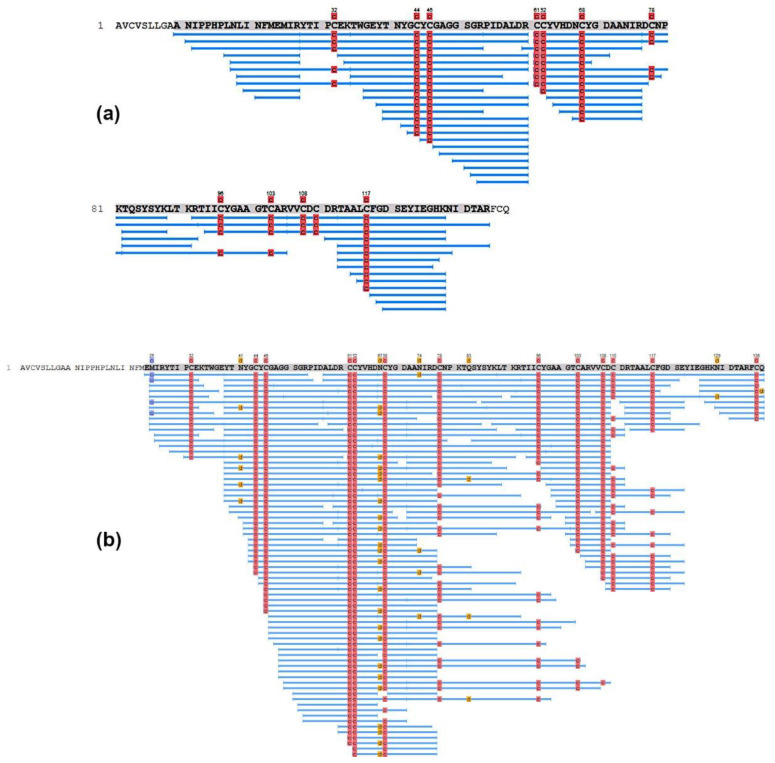
Peptide mass fingerprinting for the protein of fraction IV-18-2. The peptides found in tryptic (**a**) and Glu-C (**b**) digests and matching the shown amino acid sequence are indicated by blue lines. C on the red background shows carbamidomethylated cysteine residues, d on the yellow background shows aspartic acid produced by deamidation of asparagine and o on the blue background—methionine sulfoxide. The amino acid sequence is for the A-chain (gi|6523113) precursors of β-Bgt.

**Figure 8:**
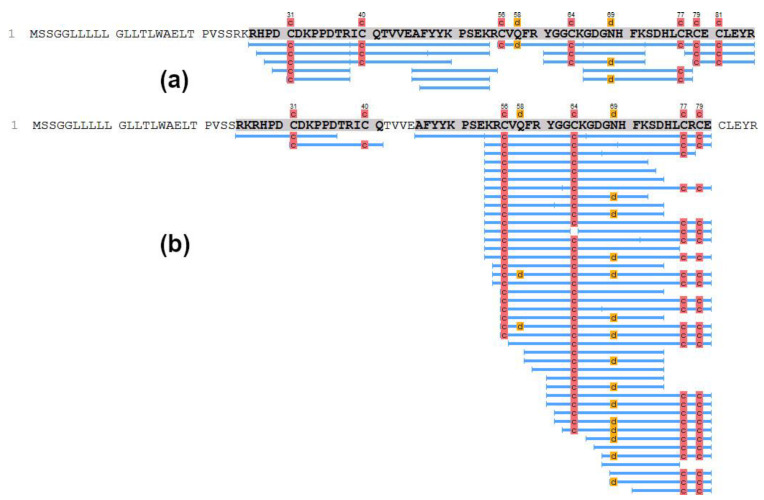
Peptide mass fingerprinting for the protein of fraction IV-18-2. The peptides found in tryptic (**a**) and Glu-C (**b**) digests and matching the shown amino acid sequence are indicated by blue lines. C on the red background shows carbamidomethylated cysteine residues and d on the yellow background shows aspartic acid produced by deamidation of asparagine. The amino acid sequence is for the B-chain (gi|82207097) precursors of β-Bgt, in which Arg45 was replaced by Glu and Asn67 by Asp.

Overall, the molecular mass of toxin IV-18-2 was 20,681.48 Da, as established by HR MS (Figure 6). The estimated value is close to that determined by MALDI-TOF MS, although is somewhat different (Table 1). In the HR MS spectrum of compound reduced with tris(2-carboxyethyl)phosphine (TCEP), only signals corresponding to B-chain were observed (Figure S1). This may be explained by the fact that the B-chains are more basic, ionized more easily and suppress the ionization of A-chains. However, these data allowed for us to calculate the precise mass of B-chain (Table 1). For the analysis of the amino acid sequence, the toxin was reduced with TCEP, carbamidomethylated with 2-chloroacetamide, hydrolyzed either with trypsin or with endoproteinase Glu-C, and the peptides obtained were analyzed. The results of LC-MS/MS analysis confirmed the assumption that the analyzed compound is a β-Bgt. In the digest of A-chain, the peptides covering the complete sequence of β-Bgt A3 chain (gi|82206358) were detected (Figure 7). Concerning B-chain, it was not possible to find any known amino acid sequence corresponding by mass and peptides to that in our toxin. We obtained a good match with MS data only with the replacement of two amino acid residues in the known B-chain (gi|82207097) (Figure 8). In the new sequence, Arg 45 in the known B-chain (gi|82207097) was replaced by Glu and Asn67 by Asp. The correctness of the new sequence was confirmed by MS-MS sequencing of the corresponding peptides (Figure S2). Thus, our toxin represents a new isoform of β-Bgt.

### 3.3. Influence of β-Bgt Modification with p-Bromophenacyl Bbromide on Its Antiprotozoal Activity

**Figure 9:**
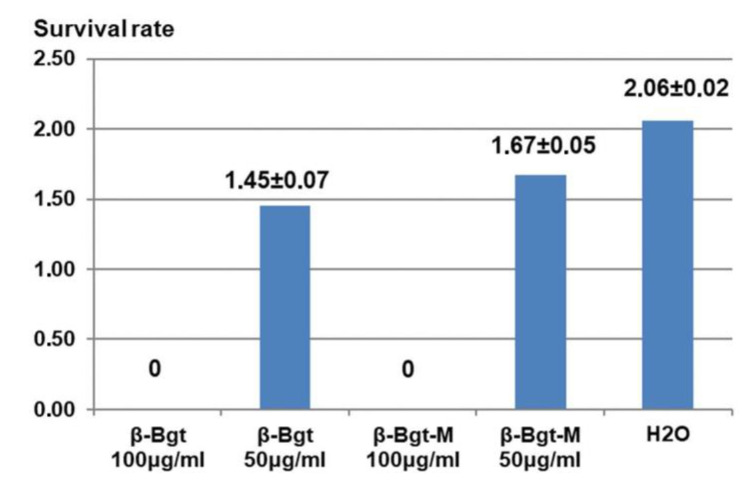
Toxicity to the *Tetrahymena* of fraction IV-18-2 (β-Bgt) and modified toxin (β-Bgt-M) in 24 h experiment. Survival rate is the ratio of the number of live ciliates after 24 h of incubation to the number of ciliates at the beginning of the experiment. Three independent measurements were performed for each concentration.

To inhibit the phospholipolytic activity of A-chain, the histidine residue in the active center of β-Bgt was modified with p-bromophenacyl bromide. The modified toxin was isolated from the reaction mixture by reversed-phase HPLC and analyzed by MS. The yield of the modified toxin was 58%. Analysis of the modified toxin by MS showed an increase in the mass by 197.97 Da, which corresponds to the incorporation of one p-bromophenacyl residue in the toxin molecule. MS analysis of the reduced modified compound showed that B-chain was not modified; therefore, p-bromophenacyl residue was incorporated in A-chain. The phospholipolytic activity of the modified toxin was determined using a sensitive fluorescence assay [45]. The phospholipase activity of native β-Bgt was very low, being equal to about 1 nmol of substrate per minute per mg of toxin, which is in good agreement with the earlier published data [45]. This value is about 3–5 orders of magnitude lower than that of phospholipases A2 from cobra and viper venoms. After modification, the activity of modified toxin decreased to a level that was practically indistinguishable from the basic spontaneous hydrolysis of the substrate. It can be estimated that the phospholipolytic activity decreased by at least an order of magnitude. However, the antiprotozoal activity after modification remained practically unchanged (Figure 9). In the 24 h experiment, both native and modified toxins killed all cells at 100 µg/mL. At 50 µg/mL, the difference between the two toxins in reducing survival rates was not statistically significant. These data mean that the phospholipolytic activity of β-Bgt is not essential for antiprotozoal activity.

### 3.4. Influence of Tetraethylammonium (TEA) on Tetrahymena Survival and Motility

We found that 100 mM TEA in the 24 h experiment reduced the survival rate to 0.69 ± 0.06 as compared to 2.06 ± 0.02 in control. With the addition of TEA, a decrease in the speed of movement of ciliates was visually noticed, and the movement changed from translational at the beginning of the experiment to rotational around its own axis. After 15–20 min, the movement reestablished. The number of alive cells decreased within an hour of observation. These data suggest that voltage-gated potassium channels may be involved in the life activity of *Tetrahymena.*

## 4. Discussion

We showed earlier that cobra venom possessed high antiprotozoal activity, which was assigned to cytotoxins [14]. Our further studies demonstrated that venoms of different snake species killed a model protozoa *T. pyriformis* with varying effectiveness [15]. Cobra venoms were the most toxic (completely killed the cells at 1 μg/mL) followed by the venom of krait *B. multicinctus* (10 μg/mL). Interestingly, the venom of another krait *B. fasciatus* was 20 times less active (around 200 μg/mL) [15]. It should be mentioned that earlier antileishmanial activity was demonstrated for krait *B. caeruleus* venom [47]. As no toxins possessing antiprotozoal activity had been isolated from krait venoms, we chose that of *B. multicinctus* for further study and isolation of active compound(s).

### 4.1. Isolation and Structural Characterization of the Active Compound

The venoms of snakes and kraits, in particular, are complex mixtures of proteins and peptides. A single venom may contain over a hundred different components belonging to different toxin families. Previously, we carried a proteomic analysis of Vietnamese *B. multicinctus* venom [42] and showed that it contained toxins belonging to 17 families. In this venom, β-Bgts were the most abundant, representing almost half (45%) of the proteins by weight, followed by three-finger toxins (28%) and phospholipases A2 (16%); other proteins were present at the level of 1–3% [42]. Each toxin family was represented by several individual toxins. For example, 19 individual three finger-toxins were identified, α-bungarotoxin being the most abundant. To isolate individual components from such a complex mixture, several types of liquid chromatography were required in the present work. We used three different types of chromatography: gel filtration, which allows for the fractionation of proteins by molecular size; ion exchange chromatography, which separates proteins by charge; and reverse-phase chromatography, which separates proteins based on their hydrophilicity/hydrophobicity. It should be noted that even when using several stages of chromatography, it is not always possible to obtain pure individual toxins, since there are such amino acid substitutions that practically do not affect the chromatographic properties of toxins, for example, Leu/Ile or Ser/Thr. Analysis of the biological activity of the obtained fractions at each chromatographic stage made it possible to identify in the *B. multicinctus* venom the component responsible for its toxicity to *T. pyriformis*.

The amino acid sequence of isolated protein was analyzed by MS, including HR MS. The latter allows for us to deduce peptide sequences directly from the experimental tandem mass spectrometry spectra [48]. The performed analysis clearly indicated that the isolated compound is β-Bgt. Structurally, β-Bgts from krait venoms are disulfide-bound dimers composed of A-chain, homologous to secretory phospholipase A_2_ (PLA2) of group I, and B-chain, a homologue of Kunitz-type serine protease inhibitor (KSPi) belonging to the Kunitz/BPTI protein family.

β-Bgts are represented in the krait venom by several structurally close isoforms in which amino acid sequences of the subunits are slightly different. These differences depend on the place of snake origin. According to the data currently available in the UniProtKB database, the amino acid sequences of at least 24 isoforms of A-chain and of 19 isoforms of B-chain are known for kraits, and the variety of possible full-size β-Bgt isoforms is likely to be even higher. In 1995, Chu et al. isolated more than 16 isoforms of β-Bgt from a batch of *B. multicinctus* venom [49]. Several isoforms were sequenced and studied in some detail [50,51].

We have found that the A-chain in the β-Bgt isolated in this work is the A3 chain (gi|6523113) from *B. multicinctus*. The B-chain of the isoform isolated from Vietnamese *B. multicinctus* venom does not correspond to any known one and is very close by the amino acid sequence to the B3 chain (gi|82207097) from *B. candidus*, differing from the latter by two amino acid residues. So, we have identified a new isoform of β-Bgt.

### 4.2. Antiprotozoal Activity of β-Bgt

The determination of antiprotozoal activity of the isolated β-Bgt showed that at a concentration of 200 µg/mL, it killed all the *Tetrahymena* cells within 2 h while the same effect was observed at 100 µg/mL in the 24 h experiment. As mentioned in the Introduction, several snake toxins manifested antiprotozoal activity. However, it is quite difficult to compare the activities of various toxins with each other, since they were determined using different methods and on different protozoa. Nevertheless, we would like to give a few examples. Thus, phospholipase A2 from the venom of the cobra *N. mossambica* suppressed the intraerythrocyte development of *Plasmodium falciparum*, which causes malaria, with an IC_50_ value of 0.032 ng/mL. At the same time, the IC_50_ value was not achieved even at a concentration of 100 μg/mL when the promastigote form of *L. infantum* was suppressed by phospholipase A2 BmajPLA2-II from the *Botrops marajoensis* venom [52]. Quite high activity against protozoa was demonstrated by the myotoxin crotamine from the venom of the rattlesnake *Crotalus durissus terrificus*. Crotamine strongly inhibited the growth rate of *L. amazonensis* with an IC50 of 25.65 ± 0.52 µg/mL [24]; it exhibited strong antiplasmodial activity and dose-dependently inhibited the development of the *P. falciparum* parasite with IC50 of 9.13 µg/mL [10]. It should also be noted that cysteine-rich secretory protein (CRISP) crovinin from the venom of *C. viridis viridis* showed an IC50 of 1–2 μg/mL, depending on the type of parasitic protozoan [27]. In addition, disintegrin from the venom of *Cerastes cerastes* was toxic to *L. infantum* promastigotes [23] at a concentration of 0.1 μg/mL, causing the death of 97.6% of promastigotes after 72 h [23]. Earlier, we have shown that cobra cytotoxins at a concentration of 1 mg/mL kill all *Tetrahymena* cells within several minutes [14]. We were unable to find any other data about the effects of snake toxins on the ciliates.

As discussed in the Introduction, *Tetrahymena* is a good model organism for all protozoa. Therefore, the toxicity data obtained on *Tetrahymena* may well be applied to pathogenic protozoa. We believe that both cobra cytotoxins and krait β-Bgt can exert adverse effects on protozoal pathogens.

### 4.3. The Elucidation of the Mechanism of Antiprotozoal β-Bgt Activity

It was discussed above that β-Bgts are disulfide-bound dimers composed of A- and B-chains. This structural feature of β-Bgt leads to the question of which chain, if any single one, may be responsible for the observed activity. As for the B-chain, we have been able to find only three examples of KSPi with antiprotozoal activity. These are aprotinin from bovine pancreas [53], a 55-amino acid peptide ShPI-I from sea anemone [54] and a 20 kDa KSPi homologue from seeds of the tropical legume liana *Derris trifoliata* [55]. Their antiprotozoal activity is believed to be based on their ability to inhibit serine proteases of parasites. However, there are no data about the inhibition of serine proteases by either the β-Bgt or its B-chain alone. On the other hand, the Kunitz/BPTI family includes diverse potassium channel blockers from animal venom. Interestingly, sea anemone toxin ShPI-I manifesting antitrypanosomal activity is a strong inhibitor of voltage-gated potassium channels [56]. Antimalarial drugs are known to have such an adverse effect as the potassium channel block [57]. It is still not clear whether the interaction with ion channels contributes to the antimalarial and, more broadly, to the antiprotozoal effect. However, ion channels in protozoan parasites are considered as potential targets for the novel antiprotozoal drugs [33,58]. Concerning β-Bgt, it is a well-known inhibitor of voltage-gated potassium channels [59]. Moreover, it was shown that B-chain alone possessed the capacity to block potassium channels [60]. Interestingly, there are potassium channels on *Tetrahymena* membrane [61] and these channels modulate the motility of ciliates [62]. Hence, it may be possible that the KSPi-like B-chain of β-Bgt is involved in the antiprotozoal action of the toxin. To check a possible involvement of potassium channels, we applied TEA, which is a non-specific inhibitor of voltage-gated potassium channels [63], and found that TEA in the 24 h experiment reduced the *Tetrahymena* survival rate. An instantaneous reaction of cells to an unfavorable change in the environment is a decrease in speed and/or a change in the way of movement. We observed the changes in the motility of *Tetrahymena* in the presence of TEA. As TEA is a fairly weak channel blocker, high concentrations (usually millimolar) of this substance are applied in the experiments (e.g., 100 mM [64]). β-Bgt is a more effective potassium channel blocker than TEA and may lead to more strong disturbances in *Tetrahymena* physiological processes, resulting in the death of the cells.

Considering the role of A-chain in antiprotozoal activity, we should mention that several snake venom secretory PLA2s have been shown to possess antiprotozoal activity [8,19]. For example, the antiplasmodial effect was demonstrated for rattlesnake crotoxin, a group II PLA2 composed of two subunits—an enzymatically active subunit CB and its inhibitor and modulator, subunit CA. CB alone is even more active than the entire crotoxin, suggesting phospholipolysis to be essential for antiprotozoal activity [65]. However, β-Bgt possesses very weak phospholipolytic activity [45]. At the same time, there are so-called myotoxic PLA2s in snake venoms; they bear substitution Asp49/Lys49 in their active center and thus lack enzymatic activity. Nevertheless, they do display antiprotozoal activity [66], but an enzymatically active PLA2 homologue is 15 times more potent than an antiprotozoal than the inactive one from the same venom [67]. This suggests that the phospholipolytic activity of A-chain may contribute to the antiprotozoal activity of β-Bgt.

To elucidate the role of phospholipolytic activity in the toxicity of β-Bgt to *Tetrahymena*, we modified the toxin with p-bromophenacyl bromide, which is known to be a specific inhibitor for phospholipases A2. Using MS, we found that the p-bromophenacyl group was incorporated into the A-chain. Earlier, it was shown that modified residue in this chain was histidine in the active site [68]. This modification was accompanied by the inactivation of the phospholipase activity. In our case, phospholipolytic activity decreased almost to zero, but the antiprotozoal activity remained practically unchanged. Therefore, we believe that the phospholipolytic activity of β-Bgt is not essential for antiprotozoal activity.

### 4.4. Future Research Directions

Considering its presynaptic neurotoxicity, β-Bgt itself, like most snake venom toxins, is unlikely to serve as a drug. However, some treatments can be utilized to reduce the general toxicity. So, in recent years, several snake toxins have been already used in different ways for the development of experimental antiprotozoal medicines. For instance, one of the ways is to enhance the effect of a known drug using its combination with a toxin. Thus, crotamine, a small myotoxin from rattlesnake venom, enhances the antileishmanial action of an antifungal antibiotic amphotericin B and reduces its cytotoxicity in vitro [69]. Another way is to utilize a special delivery system for a toxin to reduce its general toxic effect. So, a PLA2 from *B. jararacussu* venom encapsulated in liposomes displays therapeutic effectiveness comparable to that of known antiparasitic Glucantime in a cutaneous leishmaniasis mouse model [70]. A fairly original construct has been developed based on the expression of a PLA2 from the *B. pauloensis* venom in a leishmanial “suicidal” strain, which leads to the inducible death of the parasite cells in vitro [71]. The approach that also should be mentioned here is to identify in the toxin molecule the fragments of amino acid sequence responsible for interaction with the target in protozoa, followed by the synthesis of these relatively short peptides and their further use as antiprotozoals. So, a decapeptide generated from the sequence of metalloprotease BmooMPα-I from *B. moojeni* venom is an inhibitor of the purine nucleoside phosphorylase of *Plasmodium falciparum*, an enzyme necessary for the survival of the parasite [21].

The discovery of the new type of antiprotozoal toxins described in this work expands the possibilities for the design of new antiprotozoal drugs, especially if its acute toxicity is reduced by the methods discussed above.

## 5. Conclusions

In this work, we identified the new β-Bgt isoform as a main component responsible for the toxicity of *B. multicinctus* venom to the ciliate. This is the first indication of antiprotozoal activity for the class of β-bungarotoxins, and the identified toxin expands the array of snake venom proteins which possess such type of activity. The content of β-Bgts in the *B. multicinctus* venom is about 60 times higher than in the *B. fasciatus* one [42]. So, our findings in general can explain the almost 20-fold difference in the antiprotozoal activity between these two venoms [15].

Our study indicates that phospholipolytic activity is not involved in the antiprotozoal β-Bgt effect. Based on the data obtained for TEA, we suggest that β-Bgt may exert its toxicity through the inhibition of voltage-gated potassium channels.

## Figures and Tables

**Table 1:** Molecular masses of toxin IV-18-2 subunits (Da).

Chain	Molecular Masses	
	HR ^1^	MALDI ^2^
B-chain, this work	7264.52 ^3^	7264
B-chain, known	7292.48 ^3^ gi|82207097 ^4^ Mono ^5^	7291.35 gi|82207097 Av ^6^
A-chain, this work		13,408
A-chain, calculated from HR data ^7^	13,416.96	
A-chain, known	13,417.02 gi|82206358 Mono	13,426.16 gi|82206358 Av
Native compound	20,681.48	20,672

^1^ Molecular masses determined by high resolution mass spectrometry. ^2^ Molecular masses determined by MALDI TOF mass spectrometry. ^3^ In reduced form. ^4^ NCBI accession number. ^5^ Monoisotopic masses. ^6^ Average masses. ^7^ Molecular mass calculated as a difference between the mass of native toxin and the mass of B-chain.

## Data Availability

All data obtained in this study are contained within the article.

## Supplementary Materials

The following supporting information can be downloaded at: https://www.mdpi.com/article/10.3390/biomedicines11041115/s1, Figure S1: High resolution mass spectrum of B-chain; Figure S2: MS-MS sequencing of tryptic peptides containing the proposed replacement Arg/Glu45 (**a**) and N/D67 (**b**).
