# Supplementary material for: The Potassium Channel Blocker β-Bungarotoxin from the Krait Bungarus multicinctus Venom Manifests Antiprotozoal Activity
Source: Biomedicines. 2023 Apr 7;11(4):1115. doi: 10.3390/biomedicines11041115 (PMC10136136; doi:10.3390/biomedicines11041115)
Supplement: Supplementary file 1 [file biomedicines-11-01115-s001.zip › biomedicines-2215407-supplementary.pdf]

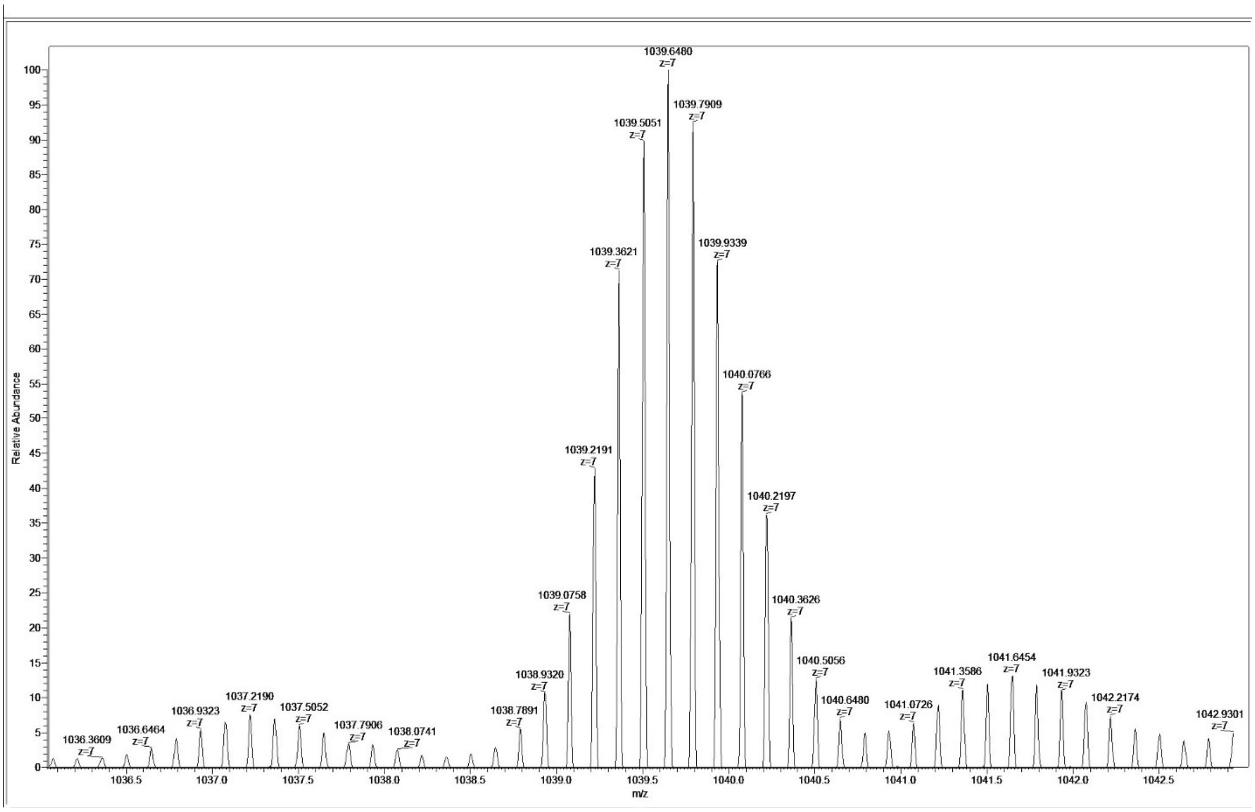

Figure S1. High resolution mass spectrum of B-chain.

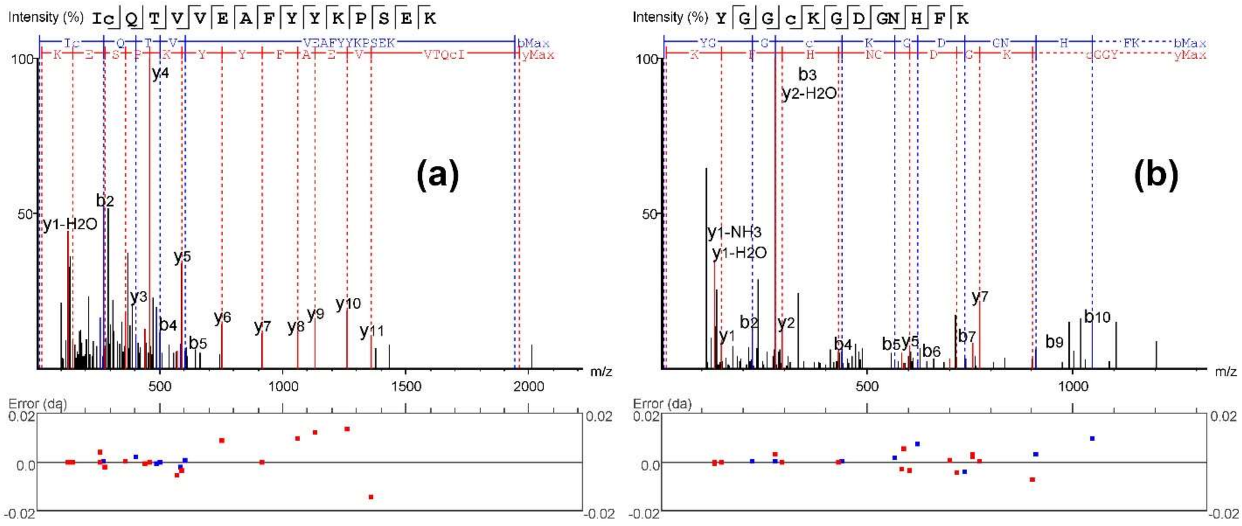

Figure S2. MS-MS sequencing of tryptic peptides containing the proposed replacement Arg/Glu45 (a) and N/D67 (b).
